# Hemangioma of the urinary bladder: an atypical location

**DOI:** 10.1590/0100-3984.2015.0231

**Published:** 2017

**Authors:** Camila Soares Moreira de Sousa, Ivo Lima Viana, Carla Lorena Vasques Mendes de Miranda, Breno Braga Bastos, Ilan Lopes Leite Mendes

**Affiliations:** 1 Medimagem, Teresina, PI, Brazil.; 2 UDI 24 horas, Teresina, PI, Brazil.

**Figure 1:**
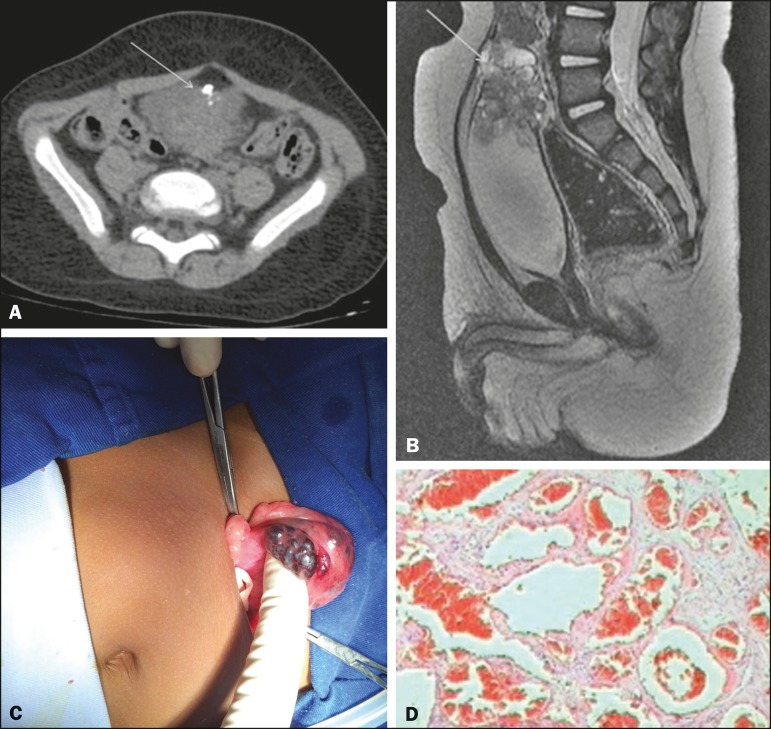
**A:** Axial computed tomography scan of the abdomen, showing a partially delimited, solid-to-cystic expansile urinary bladder lesion with a vegetative component, presenting lobulated contours, a small focal calcification, and enhancement of the solid component; the epicenter of the lesion was at the bladder dome. **B:** Sagittal reconstruction of contrast-enhanced magnetic resonance imaging of the abdomen, revealing an expansile formation, with intermediate signal intensity on T1-weighted images, heterogeneous signal intensity with a predominance of hyperintensity on T2-weighted images, the lesion showing marked enhancement. **C:** Partial cystectomy demonstrating a tumor. **D:** Histopathological section showing a lesion consistent with cavernous hemangioma.

Dear Editor,

A two-year-old male patient was referred to the pediatric emergency room with persistent gross hematuria. Abdominal ultrasound (data not shown) revealed an echogenic formation within the urinary bladder, the formation remaining fixed during changes in decubitus. Contrast-enhanced computed tomography of the abdomen showed a partially delimited, solid-to-cystic expansile urinary bladder lesion with a vegetative component, presenting lobulated contours, a small focal calcification, and enhancement of the solid component; the epicenter of the lesion was at the bladder dome (Figure 1A). A subsequent contrast-enhanced magnetic resonance imaging scan of the abdomen revealed a formation with intermediate signal intensity on T1-weighted images, heterogeneous signal intensity with a predominance of hyperintensity on T2-weighted images, and marked enhancement of the lesion (Figure 1B). Partial cystectomy was performed (Figure 1C), and the histopathological analysis demonstrated a lesion characterized by proliferation of vein-like vessels of different calibers, with intense congestion and without atypia, consistent with cavernous hemangioma (Figure 1B).

Hemangiomas are benign tumor formations of capillaries and blood vessels, common in various organs; they are extremely rare in the urinary bladder, accounting for only 0.6% of all urinary bladder tumors^(1,2)^. There have been fewer than 100 reported cases of histologically proven hemangioma of the urinary bladder^(1)^.

Most urinary bladder hemangiomas are solitary and smaller than 3 cm in diameter, affecting the dome, posterior wall, or trigone of the bladder. Although hemangiomas can occur in individuals of any age, they are seen most often in individuals under 30 years of age and are slightly more common among males^(2)^. A hemangioma usually presents as an incidental finding during the investigation of hematuria. The most common symptom is gross hematuria, which can be accompanied by irritative urinary symptoms and abdominal pain. Urinary bladder hemangiomas occasionally coexist with cutaneous hemangioma or are associated with one of two conditions^(3-5)^: Sturge-Weber syndrome and Klippel-Trenaunay-Weber syndrome.

In young patients, endoscopic findings of a bluish, sessile mass and gross hematuria are highly suggestive of hemangioma^(1)^. The main differential diagnoses for pigmented lesions seen on endoscopy include endometriosis, melanoma, and sarcoma^(6)^. Imaging tests, such as ultrasound, computed tomography, and magnetic resonance imaging, are useful in defining the location and extent of a hemangioma^(2)^.

For individuals with hemangioma, the treatment is controversial. Although there are many options available, partial cystectomy is currently the most widely used treatment for hemangioma of the urinary bladder^(3,6,7)^. Although hemangioma has a benign course, follow-up is mandatory in order to detect recurrence or residual disease^(3,7,8)^. The purpose of this case report was to underscore the importance of early diagnosis of hemangioma of the urinary bladder and of differentiating it from malignant neoplasms, which would affect the therapeutic strategy and patient survival.
